# Healthcare financing in Egypt: a systematic literature review.

**DOI:** 10.1186/s42506-021-00089-8

**Published:** 2022-01-07

**Authors:** Ahmad Fasseeh, Baher ElEzbawy, Wessam Adly, Rawda ElShahawy, Mohsen George, Sherif Abaza, Amr ElShalakani, Zoltán Kaló

**Affiliations:** 1 Syreon Middle East, Alexandria, Egypt; 2 Eötvös Loránd University University, Budapest, Hungary; 3 The School of Global Affairs and Public Policy, American University in Cairo, Cairo, Egypt; 4 Universal Health Insurance Authority, Cairo, Egypt; 5 Health Insurance Organization, Cairo, Egypt; 6 Health, Nutrition, and Population Global Practice - World Bank, Cairo, Egypt; 7 Semmelewis University, Budapest, Hungary; 8 Syreon Research Institute, Budapest, Hungary

**Keywords:** Egypt, Healthcare financing, Health system, Health insurance, Healthcare system, Health expenditure, Healthcare budget, Total health expenditure, Out-of-pocket payments

## Abstract

**Background:**

The Egyptian healthcare system has multiple stakeholders, including a wide range of public and private healthcare providers and several financing agents. This study sheds light on the healthcare system’s financing mechanisms and the flow of funds in Egypt. It also explores the expected challenges facing the system with the upcoming changes.

**Methods:**

We conducted a systematic review of relevant papers through the PubMed and Scopus search engines, in addition to searching gray literature through the ISPOR presentations database and the Google search engine. Articles related to Egypt’s healthcare system financing from 2009 to 2019 were chosen for full-text review. Data were aggregated to estimate budgets and financing routes.

**Results:**

We analyzed the data of 56 out of 454 identified records. Governmental health expenditure represented approximately one-third of the total health expenditure (THE). Total health expenditure as a percent of gross domestic product (GDP) was almost stagnant in the last 12 years, with a median of 5.5%. The primary healthcare financing source is out-of-pocket (OOP) expenditure, representing more than 60% of THE, followed by government spending through the Ministry of Finance, around 37% of THE. The pharmaceutical expenditure as a percent of THE ranged from 26.0 to 37.0%.

**Conclusions:**

Although THE as an absolute number is increasing, total health expenditure as a percentage of GDP is declining. The Egyptian healthcare market is based mainly on OOP expenditures and the next period anticipates a shift toward more public spending after Universal Health Insurance gets implemented.

## Background

Egypt is a populous African country with a population of about 102 million people in 2020 [1]. According to the World Bank classification, Egypt is one of the lower-middle-income countries (LMIC), with a Gross Domestic Product (GDP) per capita of 3100 USD in 2019 [1–3]. The Egyptian healthcare system has multiple stakeholders. It consists of a wide range of public and private healthcare providers, financing agents, and financing sources [4]. Egypt has thousands of health facilities, with about 95% of Egyptians living within 5 km of a health facility [5].

Egypt has achieved positive steps toward improving the health status of its population over the last decades. The Egyptian population became healthier over the past 20 years, and the overall life expectancy has increased from 64.5 to 70.5 years [6].

Few peer-reviewed papers discuss healthcare financing in Egypt, and there is incomplete or uncertain data on this topic. In 2004 and 2005, some studies discussed healthcare financing in Egypt [7, 8]. One study estimated out-of-pocket payments to represent 90% of the healthcare expenditure, and the Ministry of Finance and the Social Insurance Organization contributed to the remaining 10% [7]. However, this data might be outdated because several changes have occurred to the healthcare system since that time. To the best of our knowledge, after 2005, no studies focused on healthcare financing in Egypt but instead scattered data about healthcare financing in different studies.

Egypt’s healthcare system is facing extreme changes. It requires stakeholders to have a better understanding of the existing structure and what lies ahead. This study sheds light on the healthcare system financing mechanisms and the flow of funds in the Egyptian healthcare market. It also explores the anticipated challenges facing the system and the changes to come.

Our objective is to compile the available data about healthcare financing in Egypt to obtain a comprehensive overview of the healthcare financing system in Egypt. We clarify who pays for healthcare, the share of different sectors in financing healthcare, and the roles of payers and providers in the Egyptian healthcare system. It should help decision-makers set priorities and make better decisions upon understanding the structure of the system.

## Methods

We conducted a systematic literature review to find all the data related to healthcare financing in Egypt. Information about healthcare financing in Egypt was collected and analyzed from the available peer-reviewed publications and gray literature. We followed the PRISMA guidelines for reporting the SLR.

### Search strategy

The search domains were “Egypt,” “Health,” and “Financing.” These were searched using the Scopus and PubMed search engines, mainly for peer-reviewed publications. Due to the scarcity of peer-reviewed articles in Egypt on the investigated topic, we used the ISPOR’s presentations database, and Google™ search engine to search for gray literature. Appendix Table 2 shows the search terms used for different sources.

We selected articles on Egypt’s healthcare financing between 2009 and 2019 for a full-text review. We used a snowball method to identify further relevant studies among the references of full-text papers. We included all relevant articles in the review. With Google, a similar search using the keywords Egypt, health, and financing, was done, limiting the results to PDF files where the first 100 hits only were screened.

### Title and abstract screening

Two independent researchers performed the initial publications screening based on titles and abstracts, and a principal researcher resolved the disagreements between reviewers. The titles and abstracts of the search results were downloaded and imported into the EndNote citation manager (EndNote X9). Due to the overlap of coverage among the databases, the search results were de-duplicated first. Exclusion criteria were built in hierarchical order per the following: (1) No English abstract: articles with irrelevant titles and without English abstracts; (2) Unrelated specifically to Egypt: studies that are unrelated to Egypt; (3) Unrelated to human healthcare; (4) Unrelated to healthcare system financing.

### Data extraction

The same exclusion criteria applied to the full-text screening of the papers. After screening the title and abstract, one researcher extracted the data from the full text, and then, it was double-checked by another researcher. Key themes were extracted and grouped into different categories as follows: (1) General study data (author name/year/publication type/title/objective/conclusion); (2) Health expenditure in Egypt (total health expenditure (THE) as a percentage of the GDP/total health expenditure as a value in billions/governmental/public health expenditure/OOP as a percentage of THE/pharmaceutical expenditure as a percentage of THE); (3) Private health insurance (percentage covered by private insurance/private insurance type schemes available); (4) Health insurance payers and their role; (5) Healthcare system budgets (pharmaceuticals budget/medical devices budget).

### Statistical analysis

After extracting the previous data from full texts and excluding non-relevant articles, summary statistics for most data extraction categories, including maximum, minimum, median, and mean values, were calculated using Microsoft Excel. The values for the most recent year were reported, and we used the mean in cases where there was more than one value.

### Cost adjustment

Some per-patient costs were presented and adjusted to population-level using the corresponding population size in the year the data was reported based on the World Bank population data [9]. Values reported in United States dollars (USD) converted to Egyptian pounds (EGP) at the year of data reported, using the average-through-year exchange rate based on the Central Bank of Egypt data [10]. For comparing results from different years, the values were adjusted according to the consumer price index from the World Bank for Egypt [11]. If an article used a year range, the end of the range was used. Moreover, if “more than” or “less than” were used in the full text, the exact value was used.

## Results

### Summary about included papers

**Fig. 1:**
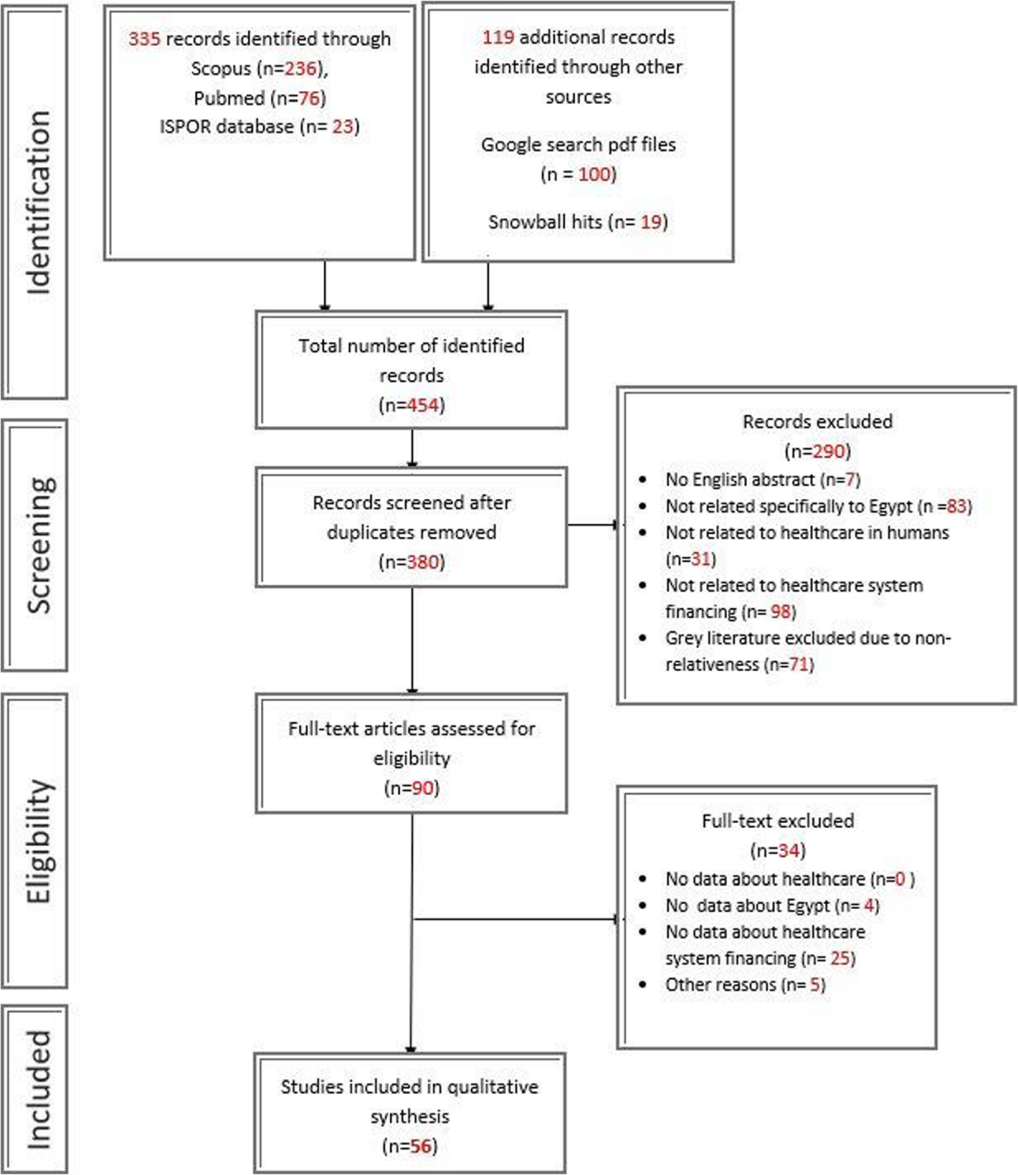
(PRISMA Diagram)

Of the total 454 records identified, 335 came from databases: 236 from Scopus, 76 from PubMed, and 23 from the ISPOR database. Furthermore, 119 records were identified from other sources: 100 from Google search pdf files and 19 from snowball hits. A total of 380 records remained after deduplication and then screened. At the end of the screening phase, 90 articles were considered eligible. Of these, we excluded 34 studies at the full-text evaluation phase for matching any of the exclusion criteria. Finally, we included 56 records in the data extraction and data analysis phase. The detailed selection process with the flow of information during the search and screening phases is illustrated in Fig. 1. Various publication types were included: 17 journal articles, six posters, 12 official reports, one book, and 20 gray literature records.

### Common objectives

Numerous studies discussed the healthcare system structure and the impact of implementing the Universal Health Coverage (UHC) from different perspectives [5, 12–20]. Other publications were about social justice in healthcare and compared the current health insurance scheme in Egypt to other countries [6, 21–26]. Many articles and official reports evaluated healthcare financing in Egypt and the expenditure pattern from governmental and household perspectives (OOP) [24, 27–37]. Several articles discussed affordability of drugs and pricing strategies that affected the drug availability in particular after currency devaluation in 2016 [38–44]. Few articles highlighted the role of private health insurance after UHC implementation and the potential opportunities to support the new insurance system [14, 45–47]. One report discussed the regional health financing landscape and provided an overview of the private health sector landscape [48].

### Country level

#### Total Health Expenditure (THE) as a percentage of GDP and as a value

**Fig. 2:**
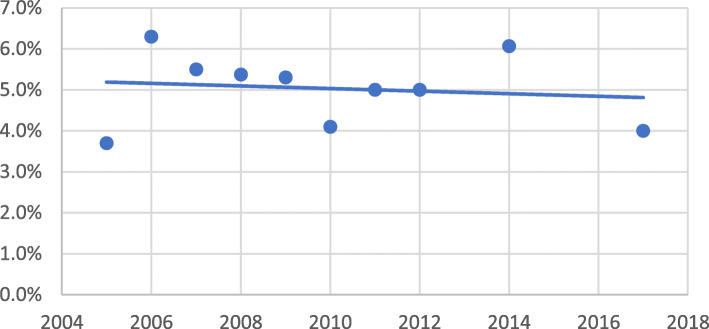
Total health expenditure as a percentage of GDP in EGYPT

THE as a percentage of GDP was almost stagnant, if not decreasing over 12 years, as shown in Fig. 2. It ranged from 3.0% [5] to 7.0% [47]. The mean was 5%, and the median was slightly higher (5.5%), while the most recently reported value average was 4% in 2017 [5, 6, 18]. In contrast, THE as an absolute value was increasing, ranging from 139 [19] to 393 [49] billion EGP. The mean and median were 222, 197 [33] billion EGP, respectively.

#### Governmental/public health expenditure

Governmental Health Expenditure (GHE) was approximately one-third of the THE, ranging from 24.8% [44] to 50% [47]. The mean was almost similar to the median (37%), and the latest reported value in 2016 was 31.2% [50]. GHE as a percent of GDP ranged from 1.8% [21] to 7.3% [51], the mean was 3.3%, the median was 2.3% while the most recent reported values’ average was 3.0% in 2014 [47, 48]. Additionally, GHE as a percent of the government budget was 6.8% on average, while the median was 5.6% [20] which was closer to the minimum 4.3% [35]. The most recent reported value in 2017 was 8.7% [6]. In contrast, the maximum average was extremely high (24%) [47]—most probably differently calculated—and it was considered an outlier. Table 1 includes data about the differences in GHE concerning the THE, the GDP, and government budget. GHE as a value in billions increased over the years. It ranged from 39 [31] to 111 [6] billion EGP, where the mean and the median were about 74 and 75 billion EGP, respectively. The most recent reported value in 2017 was 111 billion EGP [6]. According to the MoF, the government health expenditure in 2019–2020 was about 73 billion EGP [53]. Fluctuation of the reported values might be partially due to the difference in definition and the calculation methods of government and public health expenditures between articles.

**Table 1:** Governmental health expenditure as a percent of total health expenditure—GDP—government budget and absolute (adjusted to 2019) in Egypt

	GHE as a % of THE	GHE as a % of GDP	GHE as a % of GE	GHE in billion EGP
**Minimum**	24.8 [33, 52]	1.8 [21]	4.3 [35]	38.8 [31]
**Mean**	36.0	3.3	6.8	73.99
**Median**	37.8	2.3	5.6 [20]	74.97
**Maximum**	50 [47]	7.3 [51]	24 [47]	111.4 [6]
**Most recent value**	31.2 [50]	3 [47, 48]	8.7 [6]	111.4 [6]

*GHE* governmental health expenditure, *THE* total health expenditure, *GDP* gross domestic product, *GE* governmental expenditure, *EGP* Egyptian pounds

#### Pharmaceutical expenditure

The pharmaceutical expenditure as a percent of THE ranged from 26.0% [33] to 37.0% [31]. The median was 34% [43], and the mean was similar to the most recent reported value in 2011 (about 32.5%) [6]. Pharmaceutical expenditure as a value after adjustment to 2019 EGP ranged from 40 billion EGP [54] to 67 billion EGP [33]. Notably, the pharmaceutical expenditure represented about 43% [55] of OOP.

### Health care sectors

The primary healthcare financing source is OOP, representing more than 60% of THE [45], then government spending through MoF, around 37% of THE. MoF is the primary funding source for the Ministry of Health and Population (MoHP) and other disparate ministries. Therefore, MoF funds nearly one-third of the total health spending in Egypt [24]. It finances 93% of the MoHP activities and 72% of the Ministry of Higher Education (MoHE) healthcare activities [24]. Conversely, private agents, including private insurance, syndicate, firms, NGOs, employers (e.g., EgyptAir, The Arab Contractors, etc.), and donors represent the remaining 3%. Below are the dominant Egyptian health care sectors divided into public and private sectors with the new Universal Health Insurance.

#### Public sector

##### Ministry of health and population

The MoHP is the primary government entity responsible for providing preventive and curative services at the primary, secondary, and tertiary levels. MoHP provides subsidized services, 80% are free, and the rest require some user fees [24, 37]. The MoHP is a major and direct funder of parastatal organizations, including the Curative Care Organization (CCO) and the Teaching Hospitals and Institutes Organization (THIO) [17, 33, 37]. Aside from MoF funding, the MoHP is directly collecting funds from co-payments and user fees. Donors are funding via grants and loans for vertical programs (specific programs that focus on certain health conditions) [5, 6, 16, 24]. All uninsured citizens are eligible to use MoHP curative services [33]. They can also benefit from the Program for Treatment at the Expense of the State (PTES). PTES expenditure in 2008–2009 was 3 billion EGP [33, 43] which increased to over 7 billion in 2019 [56]. User fees collected at CCO and THIO are retained and do not flow into the national treasury [17, 33]. MoHP budget ranged from 3.3 to 4.0% of the annual government budget [19, 43, 57] and reached 15% of health financing in Egypt [17]. Also equal to 57% of general government health expenditure [47], 28% of total spending by the MoHP was on medical goods in 2011–2012 [5].

##### Curative care organization

The CCO is a non-profit governmental organization supervised by the MoHP. It provides services for employees of companies with dedicated contracts. It also covers accident cases, private patients, and a limited number of impoverished patients through MoHP grants [4]. The CCO has its facilities and relies on different sources of funds such as co-payments, general tax, and user fees [5, 24, 33]. The CCO uses separate funding pools, including subsidy pools, from the government for treating impoverished patients, user fees, Health Insurance Organization (HIO), MoHP, and private company contracts [58]. The CCO revenues focus on improving its services rather than generating profit. Different sources reported different breakdowns for CCO’s funding. However, looking at the National Health Accounts Egypt 2008/2009 by the USAID, we can see that the CCO attributes 46% of its funds from households, 29% of their funds from the CCO revenues, 4% through contracts with HIO and MoHP, and 12% from public firms [31]. In contrast, one paper highlighted that CCO does not receive any government subsidy, and hence its funding relies on its services revenues only [24].

##### Teaching hospitals and institutes organization

The THIO is separate under the Minister of Health authority with its own network of hospitals and specialized institutes. Estimates of THIO expenditure are 1.25% of the THE and 16.5% of the MoHP budget [33]. MoHP fund is considered the largest share of its resources (70.8%), followed by revenues from for-fee healthcare services to institutes and individuals (29.0%). Donations are minor and account for 0.2%. The THIO uses 69% of its funds to finance its hospitals and the remaining 31% for pharmaceuticals [24, 33]. The THIO budget comes from the MoF fund, MoHP, HIO, private firms through contracts, international donors’ grants, and direct user fees. Half of THIO’s services are free of charge [24].

##### Health insurance organization

The HIO is an independent government organization operating under the supervision of the Minister of Health. It provides compulsory insurance to most formal sector employees, allowing an opt-out strategy. Many published articles indicated that HIO coverage exceeds 50% of Egyptians. Between 1994/1995 and 2007/2008, the percentage of the population insured by the HIO increased from 35 to 55% [33]. In addition, the HIO expenditures rose three folds within 13 years, from 870 million EGP to reach 2.8 billion EGP in 2008 [33]. The increase was associated with increased beneficiaries and a 60% increase in the expenditure per beneficiary [33]. Several news sources reported a whopping jump in the HIO budget, reaching around 16 billion EGP in 2019 [59, 60]. According to Almasry Alyoum newspaper, it might have increased further to above 20 billion EGP in the fiscal year 2019–2020 [61].

The HIO expenditure represented 8% of the THE and represented 19% of the governmental budget [47]. The primary sources of funds are premiums and employer contributions. Beneficiary payments through cost-sharing and co-payments in some services accounted for 25% of the service fees. The remainder comes from national taxes, payroll taxes, tobacco earmarked tax, government subsidies of some population categories, such as school students, children under 7, and female, single-parent households [4, 12, 15, 17, 22–24, 31, 47].

More than 50% of HIO funding went to finance HIO hospitals, 19% used for purchasing pharmaceuticals, 4% for other medical goods. The HIO also buys healthcare services for its beneficiaries from non-HIO facilities: MoHP hospitals, 4.8%; dialysis centers, 3.4%; university hospitals, 3.1%; and private hospitals, 2.0% [58].

##### Ministry of higher education

The MoHE provides healthcare services through university hospitals [33]. It represented 6.38% of THE [33] and was funded through general tax by the MoF (72%), and user fees (27.6%). Donations comprise 0.4% of its budget. The MoHE uses 87 % of its funds to finance its hospitals (including everything except medication, such as infrastructure, medical devices, consumables, staff salaries) and 13% for pharmaceuticals [5, 24, 33]. The MoHE hospitals budget was reported to be more than 11 billion EGP in 2018 [62, 63].

##### Ministry of Defense and Ministry of Interior

The Ministry of Defense (MoD) and Ministry of Interior (MoI) provide services for their employees and local civilians. Each ministry has its network of healthcare facilities [33]. There were no available references that described the healthcare budget of the MoD or its contribution to the THE.

#### Private sector

##### Private medical insurance

The population percentage covered by private insurance ranged from 1% [21] to 10% [45]. The mean was 4.3%, the median was 3.0%, while the most recently reported value in 2019 was 5.0% [14].

##### Household out of pocket

**Fig. 3:**
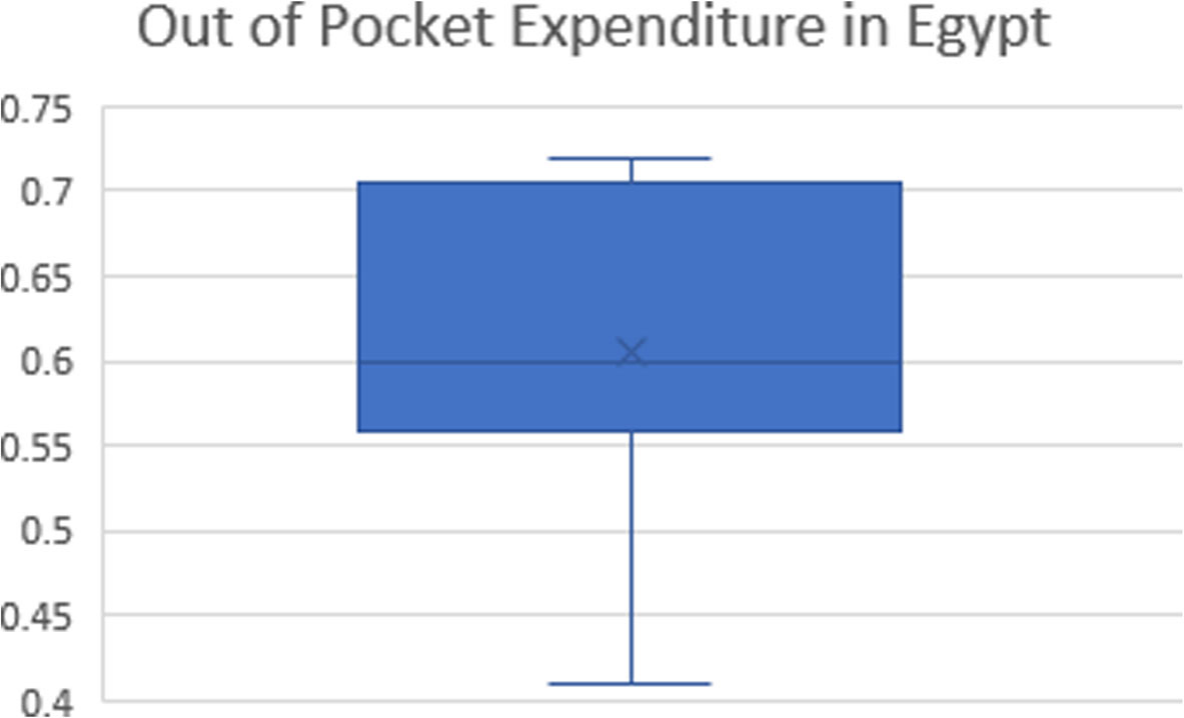
Out-of-pocket expenditure in Egypt

OOP payments are considered the largest source of healthcare financing in Egypt. It ranged from 41% [47] to 72% [55], the mean was 63%, the median was 60% [55], and the most recent reported value in 2017 was 56% [18], as demonstrated in Fig. 3. Private clinics consume the more share 38.4%, followed by pharmaceuticals at 33.1%. Concerning hospitalization services, private hospitals receive the lion’s share with 8.2%, followed by MoHP hospitals at 3.5% [33].

##### Nongovernmental organizations

Although Nongovernmental Organizations (NGOs) do not contribute significantly to the health care financing budget in Egypt, they play a role in primary healthcare services and raising public awareness [58]. About one-quarter of NGO funds come from domestic donations, and the remaining funds come from external funding sources [33].

### Universal health insurance

The UHI is a new entity established to provide Universal Health Insurance services for all Egyptians. The UHI system is financed through several sources such as citizen-paid premiums, state budget, government subsidization of the poor, general tax, tobacco earmarked tax, co-payments (service fees), a contribution of 0.25% of total annual revenues, and fees ranging between EGP 1000 and EGP 15,000 paid by hospitals, medical clinics, treatment centers, pharmacies, and pharmaceutical companies to subscribe to the new health insurance system [20, 34, 64, 65].

The new citizen-paid premiums are as follows: employers would pay a 4% premium of each employee’s salary into the fund (3% for medical insurance + 1% for work injuries and occupational diseases insurance). Employees would pay another 1% premium, which would come from their salary. In addition, breadwinners would pay premiums of an extra 1% for each dependent and 3% for housewives, sanctioning all family members to be insured. The state is responsible for the costs of treatment of those who are unable to be fully determined by the Ministry of Social Solidarity according to the Prime Minister decree number 1948 in 2019 [66].

The cost of the Universal Health Insurance for one citizen will range from EGP 1300 to EGP 4,000, from a mere figure of EGP112 in the current insurance system [64], in line with Egypt’s macroeconomic target to increase spending on health, education, and research and development to at least 10% of GDP [67].

Provision of the comprehensive basic package will be based on competition and choice among the different public and private service providers, under a single public and health insurance fund (PHIF) using incentive-based and other provider payment mechanisms [5].

## Discussion

Egypt currently has a multiparty tangled healthcare system with several disparate public and private providers and fragmented financing sources. The MoHP acts as one unit encompassing the financing and provider functions under one entity [4]. Similarly, HIO acts as a simultaneous payer and service provider, which affects the quality of services provided. Egyptians who have a higher ability to pay usually utilize private sector facilities and pay out of pocket [68].

One of the significant issues in the Egyptian healthcare financing system is the gigantic out-of-pocket proportion which puts families at a high financial risk of catastrophic expenditure and decreases the performance of the healthcare system. Catastrophic health expenditure is caused primarily by out-of-pocket payments that lead to households falling below the poverty line [69, 70]. More than 20% of Egyptians encounter catastrophic health expenditures. It is higher than the corresponding values of catastrophic payments in low-middle income countries like India or Bangladesh. The trend continues to rise over time toward even more catastrophic health expenditure in Egypt [36, 71].

Therefore, it was a pressing need for the Egyptian government to seek Universal Health Insurance (UHI) to ensure available healthcare services to the whole population regardless of their income level. As a response, the government issued law number 2 for 2018 [66], which stated the establishment of the new health insurance system in line with Sustainable Development Goal number three [72] and the Sustainable Development Strategy for Egypt (Egypt vision 2030) [73].

UHI systems work in most countries with efficient or satisfactory health systems, like Germany, France, Canada, and the UK [74]. China has started a healthcare system reform in 2009 toward UHC in 2030. Their experience shows that the road toward full implementation is tough and resource exhausting, but not impossible, even in a significant population like China [75]. Many Arab countries like Tunisia, Saudi Arabia, and Libya are also on the road to implementing UHC. However, financing remains a critical issue that hinders the process even in higher-income countries [19, 76, 77].

Health care financing efficiency can improve by increasing the proportion of public funding and reducing fragmentation of financing through implementing UHI. The new UHI will be trying to tackle the giant out-of-pocket payments and catastrophic health expenditure issues in several ways, for instance, by including all family members in the new insurance scheme and covering the poor from the state budget. Due to the lack of resources, it is hard to sufficiently finance comprehensive healthcare coverage for all Egyptians in one stage. Therefore, the implementation of the new insurance system will be through six phases ending by 2032.

When Egypt’s UHI system is complete, its budget alone will surpass the current THE, significantly increasing the THE as a percentage of GDP. The UHI is divided between the payers and providers of services. Furthermore, providers will no more be dominantly public providers. Instead, all providers can enroll under the umbrella of the new system. The payer-provider split has worked in several countries with goals of cost containment, better efficiency, improved responsiveness to needs, and the creation of competition between different providers [78].

A healthy competition between health care providers usually leads to better quality care. It strengthens the patient position concerning providers. It is also a tool for matching the services with patient needs and allocating resources efficiently [79]. When patients are obliged to use certain health facilities, there’s no competition and, therefore, no incentive to provide better services. When patients can access better healthcare facilities, the lower quality facilities will eventually try to offer better services to attract patients and generate revenues.

Compared to countries with more developed healthcare systems, private insurance in Egypt does not currently cover a large proportion of the population. When it comes to financing, it has an even smaller share. Private health insurance (PHI) can have a new role after implementing UHI, in the form of providing complementary health insurance (CHI) and supplementary health insurance (SHI) in addition to the public health insurance scheme [14, 65, 80].

Because of the high purchasing power parity of the Egyptian pound, outpatient and healthcare services generally are cheaper than regional and global averages. Due to external price referencing, innovative pharmaceuticals have a narrower price corridor than outpatient and hospitalization services [81]. It results in pharmaceutical expenditure composing a significant part of the healthcare budget in Egypt compared to other countries, as it presents 32% of THE and 43% of household expenditures [55].

The upcoming changes in the healthcare system structure will bring several challenges, owing to the considerable budget expected for implementing UHI. The higher premiums in the new system will raise the expectations of the beneficiaries toward the quality and comprehensiveness of the provided services. Also, the split of the current unified payer-provider system will bring administrative challenges. However, experiences from other countries that have implemented a similar system may help to overcome those challenges.

### Study limitation

Obtaining accurate recent estimates for the Egyptian budgets and healthcare spending is not an easy task. The available data is outdated. The latest official data is usually from 2016 or before. Devaluation occurred in Egypt in 2016, so any data reported before 2017 are not very reliable. However, percentages may still apply. In many cases, complementary values might not sum to 100% because the extracted data came from several diverse sources.

## Conclusions

The findings of this Systematic Literature Review (SLR) provide an overview of the structure and dynamics of healthcare financing in Egypt. Few peer-reviewed papers discussed healthcare financing in Egypt, and so most of the data came from gray literature, indicating the topic is under published. The results of this SLR could improve budgeting, planning, and policymaking. Although values of the same estimate were heterogeneous between different studies, our review outlines the healthcare financing indicators and structure, funding mechanisms, budgets for disparate payers in the healthcare system. Despite the increasing THE as an absolute number, the THE as a percentage of GDP is decreasing. When the absolute numbers were adjusted for inflation, the real expenditure in billion EGP seems stagnant in the last couple of years. GHE as a percentage of GDP ranged from 3% [5] to 7% [47], with the two most recent references reporting 3% [5, 6]. According to the parliament obligation in 2014, GHE should not be less than 3% and should gradually increase to match the global levels of THE as a percentage of GDP, which is around 10% [82, 83]. Out-of-pocket expenditures on healthcare services are seemingly still huge, representing about two-thirds of the THE in Egypt. The implementation of UHI will hopefully decrease this percentage by driving the market to a more governmentally funded system, decreasing catastrophic health expenditure for citizens.

## Data Availability

All data generated or analyzed during this study are included in this published article [and its supplementary information files]. Data used in this study are available upon reasonable request.

## Appendix

**Table 2:** Search strategies and number of hits for healthcare financing in Egypt in different databases

Source no.	Search area	Search	Date of search	Number of hits
PubMed (Medline)				
#1	Combined search	((((Egypt[Title/Abstract]) AND ((health[Title/Abstract] OR medica*[Title/Abstract] OR medici*[Title/Abstract]OR pharmaceu*[Title/Abstract]))) AND ((finan*[Title/Abstract] OR budget[Title/Abstract] OR expen*[Title/Abstract]))) AND (“2009/12/05”[Date - Publication] : “3000”[Date - Publication]))	5/12/2019	76
SCOPUS				
#1	Combined search	(TITLE-ABS-KEY (Egypt) AND TITLE-ABS-KEY (health OR medic* OR pharmac* ) AND TITLE-ABS-KEY ( financ* OR budget OR expen* ) ) AND PUBYEAR > 2008	5/12/2019	236
ISPOR				
#1	Scientific presentations database	Egypt AND (health OR medic* OR pharmac*) AND (financ* OR budget OR expen*) *Note: in topics Health Policy & Regulatory OR Health Technology Assessment*	5/12/2019	23
Total				335
Google				
	Google search engine	“Egypt” and Health care and finan* filetype:pdf	08/12/2019	7,700,000 (First 100 were screened) #

## Abbreviations

CCO: Curative care organization
CHI: Complementary health insurance
EGP: Egyptian pounds
GDP: Gross domestic product
GE: Governmental expenditure
GHE: Governmental health expenditure
HIO: Health insurance organization
LMIC: Lower-middle-income countries
MoD: Ministry of Defense
MoHE: Ministry of Higher Education
MoI: Ministry of Interior
MoF: Ministry of Finance
MoHP: Ministry of Health and Population
NGOs: Nongovernmental organizations
OOP: Out of pocket
PHI: Private health insurance
PHIF: Public and health insurance fund
PTES: Program for treatment at the expense of the state
SHI: Supplementary health insurance
SLR: Systematic literature review
THE: Total health expenditure
THIO: Teaching hospitals and institutes organization
UHC: Universal health coverage
UHI: Universal health insurance
USD: United States dollars
